# Corticosteroid Use in Musculoskeletal and Neuraxial Interventions: Effects on Glycemic Control

**DOI:** 10.3390/medicina61050936

**Published:** 2025-05-21

**Authors:** Brian Singer, Dovid Chaimovitz, Thomas Bucek, Eli Dayon, Aimee Abbott-Korumi, Moshe Spatz, Tejaswi Makkapati, Hayk Petrosyan, Laurent Delavaux

**Affiliations:** 1 Department of Physical Medicine and Rehabilitation, JFK Johnson Rehabilitation Institute at Hackensack Meridian Health, Edison, NJ 08820, USA; brian.singer@hmhn.org (B.S.); thomas.bucek1@hmhn.org (T.B.); aimee.abbottkorumi@hmhn.org (A.A.-K.); tejaswi.makkapati@hmhn.org (T.M.); hayk.petrosyan@hmhn.org (H.P.); 2 Rosalind Franklin University of Medicine and Science, Chicago, IL 60064, USA; dovid.chaimovitz@my.rfums.org; 3 Burke Rehabilitation Hospital, White Plains, NY 10605, USA; edayon@burke.org; 4 Rutgers New Jersey Medical School, Newark, NJ 07103, USA; moshe.spatz@rutgers.edu

**Keywords:** pain management, corticosteroid injections, musculoskeletal medicine, chronic pain, hyperglycemia

## Abstract

Effective multidisciplinary pain management involves an in-depth knowledge not only of diagnosis and treatment but of how interventional procedures affect patients across all health domains. One of the most common pharmacological tools utilized in patients suffering from chronic pain disorders is corticosteroids. Corticosteroids are leveraged for their anti-inflammatory properties across a wide range of disorders. This review examines the role of corticosteroids and pain management with a specific focus on their metabolic impact regarding glucose metabolism. Corticosteroids have been shown to increase gluconeogenesis, resulting in reduced insulin sensitivity and an impaired peripheral glucose uptake. These varied responses to corticosteroids are especially concerning given the high prevalence of diabetes mellitus in chronic pain patients. There is well-documented evidence of not only transient hyperglycemia but emerging literature on prolonged glycemic disturbances that may have a greater effect on patients than previously recognized. A review of the available literature reveals variations in hyperglycemia depending on corticosteroid type, dose, and various patient-specific factors. Some research does suggest that lower corticosteroid dosages can provide similar therapeutic benefits and potentially reduce glycemic aberrations. Given the current evidence, clinicians should closely monitor patients’ hemoglobin A1C levels when determining the risks and benefits of an interventional procedure and consider alternative pain management strategies when appropriate. Future research should focus on optimizing corticosteroid selection and dosing to balance the safety, particularly in diabetic or prediabetic patient populations.

## 1. Introduction

Effectively treating patients’ pain is required to improve their overall quality of life as well as enable them to perform general activities of daily living (ADLs). Pain management strategies traditionally include a multimodal approach, including physical therapies, medication management, psychological interventions, and even medical or surgical procedures for refractory cases [1,2]. Among the pharmacological options, corticosteroids have been shown to be an effective option for managing pain associated with inflammatory conditions. These drugs mimic the hormone cortisol, which has potent anti-inflammatory and immunosuppressive properties that can control pain in various conditions. Corticosteroids are generally indicated in the management of several conditions, including osteoarthritis (OA), rheumatoid arthritis (RA), radiculopathy, tendinitis, bursitis, and other inflammatory joint diseases, and can be especially useful when nonsteroidal anti-inflammatory drugs (NSAIDs) and other conservative treatments have failed [2,3].

Corticosteroids can be administered using various routes, including oral, intravenous, and intra-articular injections, based on individual patient needs [4,5]. Peripheral joint injections are one of the most common uses of corticosteroids in treating pain [6]. Corticosteroids are directly injected into an inflamed joint or surrounding tissue, which allows for high concentrations of the medication to be delivered more accurately to the site of pathology or pain [3,7,8]. This method maximizes therapeutic efficacy while minimizing the systemic side effects associated with steroids [9]. The most common injection sites include the knee, shoulder, hip, and elbow, as they are the most common areas of musculoskeletal inflammation and pain. These injections can provide long-lasting relief for patients, improve mobility, and reduce the need for oral medications [10].

Despite their beneficial therapeutic effects, corticosteroids also have significant metabolic side effects, especially in regard to glucose metabolism. Corticosteroids can raise blood sugar levels, which can be a problematic side effect for diabetic patients, whose condition is characterized by chronic hyperglycemia due to defects in insulin production or secretion [9,10]. As a result, these patients already have a dysregulated glucose metabolism and are prone to more glucose fluctuations. The use of corticosteroids in this patient population can exacerbate this problem, potentially leading to uncontrolled hyperglycemia and an increased risk of complications [11]. This paper summarizes the evidence to provide an overview of the role of corticosteroids in pain management, with a particular focus on their effects on blood glucose levels in patients.

## 2. Types of Corticosteroids and Their Clinical Applications

Corticosteroids are classified into three major groups: mineralocorticoids, glucocorticoids, and adrenal androgens. The majority of clinically therapeutic injectable solutions will utilize medications in the glucocorticoid class. The three glucocorticoids described below share a common mechanism of action; they bind to and activate a type of nuclear receptor aptly named the glucocorticoid receptor. Once bound to the receptor, glucocorticoids lead to the production of lipocortin (annexin-1), which then inhibits PLA2 and COX-1/COX-2, which decreases the overall production of leukotrienes, prostaglandins, and platelet-activating factors. The indirect effect on leukocytes involved in inflammatory events is to impair their epithelial adhesion and chemotaxis. This cascade also decreases the recruitment of more immune classes, such as macrophages, for phagocytosis to the area in question [8,12]. These varied effects on immune cells creates a potent anti-inflammatory effect via suppression of pro-inflammatory cytokine production, including interleukin-1, tumor necrosis factor, and others. This downregulation of cytokine secretion by immune cells further reduces immune cell activation. This multi-tiered mechanism of altered physiology results in decreased inflammation and reduced pain [13]. A potential pitfall of glucocorticoid utilization is the unwanted immunosuppressive results of these desired anti-inflammatory and pain-relieving effects. There are other proposed mechanisms of action that are believed to potentiate the therapeutic effects of corticosteroids, including modulation of peripheral nociceptive neurons and spinal cord dorsal horn cells; overall, these medications have a direct stabilizing effect on neural membranes as well [12].

While glucocorticoids act primarily on the glucocorticoid receptor, they also do have variable activity on nearby mineralocorticoid receptors due to their structural similarity. Mineralocorticoids, such as aldosterone, are important in the regulation of electrolytes such as sodium and potassium, which can result in disruption of major chemical processes and fluid shifts. Aldosterone affects various systems in the body, notably via blood pressure changes [14,15,16]. Corticosteroid medications vary in their degree of mineralocorticoid receptor activity due to their preference for mineralocorticoid receptors or glucocorticoid receptors [17]. Medications such as hydrocortisone and cortisone have significant mineralocorticoid activity, which makes them less ideal to use for anti-inflammatory benefits and more preferential to use in cases of adrenal insufficiency, as they mimic the effects of aldosterone [17]. Meanwhile, steroids such as methylprednisolone and dexamethasone have very minimal mineralocorticoid receptor activity due to preference for glucocorticoid receptors [17,18]. Medications such as prednisone and prednisolone have high glucocorticoid activity and low mineralocorticoid activity, which makes them excellent choices to use to combat systemic inflammation. Systemic steroids can impact mineralocorticoid receptor signaling indirectly by oversaturating the 11β-hydroxysteroid dehydrogenase enzyme that works to prevent glucocorticoid effects on cells [19]. This enzyme is located in large quantities in organs such as the kidneys and pancreas and absent in others, namely the heart and central nervous system, which can contribute to steroid side effects such as elevated blood pressure and insomnia [20].

Different corticosteroids are commonly utilized for pain management, often given as an injection. Triamcinolone and methylprednisolone (MP), for example, are widely reported in the literature as the primary corticosteroids used for musculoskeletal peripheral injections [21,22,23]. One study comparing triamcinolone and betamethasone found triamcinolone to provide a more sustained therapeutic effect [24]. Similarly, a study assessing their use in knee osteoarthritis (OA) showed that while both provided temporary symptomatic relief, triamcinolone was more effective at three weeks, whereas methylprednisolone retained efficacy further out to 8 weeks [25]. Another study evaluating intra-articular triamcinolone versus methylprednisolone found them both to provide pain reduction and improved function with a comparable duration of efficacy [21]. Despite methodological variations across studies, the conclusion can be drawn that corticosteroids commonly used for peripheral joint injections have shown comparable levels of efficacy. The solubility and composition of corticosteroids also influence their potency, efficacy, and duration of action [26,27,28,29].

Factors such as potency, duration of action, steroid particulate size, and aggregate potential are major determinants in the physician’s choice of corticosteroid. The potency of glucocorticoids is assigned a numerical score, which indicates potency relative to that of cortisol, which is given a score of “one”. There are a handful of glucocorticoid corticosteroids that are commonly used therapeutically:

### 2.1. Prednisone

Prednisone is a widely used synthetic corticosteroid that is commonly prescribed for a variety of inflammatory and autoimmune conditions [30]. Prednisone is considered an intermediate-acting steroid with a duration of action of 24 to 36 h. Prednisone is an inactive agent that is metabolized to the active agent prednisolone by the liver [9]. The typical dosage of prednisone ranges from 5 to 60 mg per day, depending on the diagnosis and severity of the condition. For chronic conditions like rheumatoid arthritis, a common dose is 5–10 mg oral, daily. For acute flare-ups or severe inflammation, higher doses may be prescribed, often starting at 40–60 mg oral per day and tapering as the patient responds to treatment [30].

### 2.2. Methylprednisolone

Methylprednisolone is another widely used corticosteroid that is available in oral and injectable forms. Methylprednisolone has a relative glucocorticoid potency of 5. The maximum particle size of methylprednisolone is quite large and is often greater than 500 μm. It does, however, have a smaller average particulate size (0.5–26 μm) compared to triamcinolone (15–60 μm). This medication also has extensive particle aggregates, which would increase the risk of use in spinal injections, secondary to unintended ischemic events occurring with accidental intra-arterial injection. Oral therapy doses typically range from 4 mg to 48 mg per day. In severe cases, intravenous administration is required, with doses ranging from 40 mg to 120 mg per day [13,26,31]. Methylprednisolone’s mechanism of action is similar to other corticosteroids. Methylprednisolone, however, is also believed to be able to more significantly suppress the migration of immune cells to the site of inflammation, which further reduces the inflammatory response [13,31].

### 2.3. Triamcinolone

Triamcinolone is one of the most commonly used synthetic corticosteroids for peripheral joint injections. A microcrystalline corticosteroid with low solubility, it exhibits greater anti-inflammatory potency and a prolonged therapeutic effect due to its remaining at the injection site longer [26]. Triamcinolone is considered an intermediate-acting steroid with a duration of action of roughly 24 to 36 h, and has a relative glucocorticoid potency of five. It is a large particulate steroid with a maximum particle size greater than 500 μm. Triamcinolone experiences extensive particle aggregation, which, when combined with the large particulate size and solubility, can increase its duration of action in the peripheral joints but pose a significant amount of risk for spinal use. Physicians utilize a wide range of dosages for triamcinolone, ranging between 5 mg and 80 mg per joint [9].

### 2.4. Dexamethasone

Dexamethasone is a potent, long-acting steroid with a duration of action longer than 48 h. It is more potent than the above medications and has a relative glucocorticoid potency of 27. Dexamethasone is regarded as a “non-particulate” steroid, as its maximum particle size is 0.5 um. Dexamethasone possesses the lowest density of the glucocorticoids and a minimal tendency to aggregate. Its high solubility contributes to a more rapid onset of action and relatively greater potency; however, this also results in a shorter duration of effect [13,29]. In clinical practice, it is often preferred for conditions that require strong immunosuppressive effects [31]. It is also used in cancer treatments to manage the side effects of chemotherapy. The typical dosage for dexamethasone ranges from 0.5 mg to 10 mg, depending on the severity of the condition. Dexamethasone can also be given in peripheral joint injections using 5–20 mg. Of note, this agent is a common choice for spinal injections, as any inadvertent intra-arterial injection of dexamethasone will not cause any downstream arterial occlusions thanks to its very small particle size.

### 2.5. Corticosteroids and Glucose Metabolism

Corticosteroids, in particular glucocorticoids, affect glucose metabolism through multiple routes. Glucocorticoids can influence acute alterations in glucose homeostasis as well as affect long-term metabolic mechanisms. Short-term effects on blood glucose are mainly mediated through three physiological aberrations: increased gluconeogenesis, development of insulin resistance, and reduced peripheral glucose uptake, as well as suppression of pancreatic function. Glucocorticoid corticosteroids upregulate hepatic gluconeogenesis pathways, resulting in hyperglycemia; this is mediated through the upregulation of key gluconeogenic enzymes, including phosphoenolpyruvate carboxykinase (PCK) and glucose-6-phosphatase. These two enzymes have been shown to be critical in gluconeogenesis, especially in the liver and kidney. Overactivation of these key enzymes leads directly to increased release of endogenous glucose into the circulation, resulting in hyperglycemia [32].

Glucocorticoids can also lead to the induction of peripheral insulin resistance. This is theorized to be due to the corticosteroid-induced changes in the expression of genes involved in glucose metabolism, namely those encoding glucose transporters as well as enzymes involved in gluconeogenesis. Namely, the downregulation of glucose transporter type 4 interferes with insulin receptor signaling cascades. The effect of this is to decrease the glucose uptake by skeletal muscle and adipose tissue, causing high circulating levels of glucose and hyperglycemia [11,30,33]. This issue can be magnified in the setting of insulin resistance, as is commonly seen in diabetic patients [34,35].

Furthermore, glucocorticoids have been shown to suppress pancreatic beta cell function, which results in a decrease in insulin secretion. Different glucocorticoids have the ability to inhibit L-type voltage-gated calcium channels found in the cell membranes of several cell populations, including the beta cells of the pancreas. Reduced calcium influx across the cell membrane of the beta cells prevents the accumulation of a sufficient calcium ion gradient required to trigger insulin exocytosis. This reduction in insulin secretion, combined with insulin resistance and increased glucose production, can result in significant corticosteroid-induced hyperglycemia in patients with diabetes [34,36]. These fluctuations may last several days, even after the steroids have been discontinued [37].

Prolonged or repeated exposure to corticosteroids can have more serious effects on glucose metabolism, which can range from acute fluctuations to persistent abnormalities. Persistent hyperglycemia is often reflected by an increase in patients’ Hemoglobin A1C (HbA1C) levels, as this blood assay represents a measure of average blood glucose levels over several months. In non-diabetic patients, long-term exposure to corticosteroids can increase their risk of developing steroid-induced diabetes [38]. This is believed to occur due to a multitude of factors. As discussed, patients can experience sustained decreased levels of insulin due to repeated suppression of pancreatic beta cells. This prolonged suppression of beta cells has been shown to lead to apoptosis of these cells and permanent loss of function. Patients also experience altered adipokine profiles. Changes to adipose tissue function and hormone secretion cause metabolic dysregulation. Increased gluconeogenesis through the previously described PCK and G6P enzymatic upregulation pathways becomes all the more impactful as glucose newly released into the serum is no longer being removed and stored at a great enough rate. In addition to uptrending HbA1C levels, patients can experience other metabolic changes as well, most commonly weight gain, dyslipidemia, and abdominal fat accumulation, which can further exacerbate insulin resistance [35,38].

## 3. Potential Local and Systemic Side Effects of Corticosteroids

Corticosteroid injections are commonly used for conditions like osteoarthritis, tendinopathy, and bursitis. Although they provide short-term pain relief, these injections carry potential risks, which are categorized as local and systemic and may occur either immediately (within 48 h) or delayed (after 48 h). These side effects particularly affect the endocrine system [39,40,41]. Available literature indicates that immediate side effects include both adrenal suppression and transient increases in blood sugar levels [39,42,43,44]. Of note, clinicians should be aware that localized adverse effects, including skin hypopigmentation and subcutaneous fat atrophy, have also been reported.

### 3.1. Effects on Blood Glucose

Transient corticosteroid-induced hyperglycemia can affect both diabetic and non-diabetic patients [39]. While serum corticosteroid levels peak within around 22–24 h, as seen in two studies of interlaminar epidural triamcinolone injection [45,46]. These hyperglycemic effects can potentially last longer than one week [39]. Unsurprisingly, these effects were found to be more pronounced in diabetic patients, with hyperglycemic effects lasting for several days to weeks [39]. Additional delayed side effects following the intraarticular administration of corticosteroids included increases in blood glucose levels that often peak 1 to 3 days after injections [47]. This can lead to hyperglycemic episodes or, even in extreme cases, ketoacidosis in patients with diabetes. These complications could potentially evolve into a life-threatening emergency. Hao et al. [43] examined patients receiving a single epidural injection of methylprednisolone at doses of 10 mg, 20 mg, and 40 mg. There was a statistical significance between serum cortisol and fasting plasma glucose between the 10 mg group and 40 mg group, suggesting a dose-dependent increase in blood glucose.

The studies note that it is recommended for patients with diabetes to monitor glucose levels closely following any such injections and that corticosteroid injections should be avoided in those poorly controlled (i.e., fasting glucose > 200–250) [40]. One retrospective study found that patients with a pre-procedural HbA1C greater than 7% who were also on insulin showed the greatest increase in post-procedural blood glucose levels [43]. Interestingly, higher hemoglobin HbA1C levels prior to injection were also associated with greater blood glucose derangements after injection, while the specific steroid used did not seem to have any bearing [43]. In one large retrospective cohort study reviewing 1169 diabetic patients receiving intra-articular corticosteroid injections, the only factor associated with a greater than expected increase in post-injection HbA1C was a baseline HbA1C over 8.0% [48].

### 3.2. Effects on Endogenous Cortisol

The risk of prolonged adrenal insufficiency is one of the delayed side effects of repeated corticosteroid injections that can impact glycemic control [49]. Corticosteroid absorption into the bloodstream following intra-articular injection can suppress the hypothalamic–pituitary axis, leading to decreased cortisol production, which can last for at least one-week post-injection. Multiple injections may increase the likelihood of systemic absorption [40,42]. In one study, patients treated with epidural injection of methylprednisolone showed residual HPA dysfunction, measured as decreased serum cortisol, in 43% of participants at week two, with all participants normalizing by week four [42]. Another work found that patients who received an epidural injection of corticosteroid plus lidocaine had significantly lower cortisol levels at the 3-week follow-up compared to those who received only lidocaine [30]. Beyond potency, the specific type or dose of corticosteroid used may have a bearing on how severe the expected cortisol suppression might be following corticosteroid injection, which may be attributable to differences in water solubilities [50]. These effects were more pronounced in diabetic patients but were not statistically significant.

Although there is no formal guidance on the frequency or maximum lifetime use of corticosteroid injections, radiologists and clinicians should ensure that patients are properly informed about the risks and the importance of monitoring for both local and systemic complications [40]. In one RCT, 84 patients with symptomatic knee osteoarthritis were treated with either 10 mg or 40 mg intra-articular triamcinolone. Interestingly, the study revealed little difference from baseline in pain visual analog scale at 12 weeks between the two doses, with the 10 mg dose deemed noninferior to the 40 mg dose [51]. A similar study looking at different doses of triamcinolone for intra-articular glenohumeral joint injections revealed no significant differences in Shoulder Pain and Disability Index (SPADI) at 6 months between 20 mg, 40 mg, or 80 mg of triamcinolone [52]. A similar evaluation of patients receiving lumbar interlaminar epidural injections showed similar pain relief at one month between those receiving 5mg dexamethasone vs. those receiving 10 mg dexamethasone [53]. Many other similar studies not included here highlight the growing awareness amongst the interventional community that using lower doses of corticosteroids for various interventions may offer similar therapeutic benefits while minimizing the deleterious effects outlined in this review. This is likely another avenue that might help guide safer care, particularly for diabetic patients in need of focused intervention.

## 4. Discussion

Corticosteroids are known to significantly impact blood glucose levels through several mechanisms [54,55]. Primarily, they promote gluconeogenesis, a metabolic pathway that results in the generation of glucose from non-carbohydrate substrates in the liver. This process is upregulated by corticosteroids, leading to increased glucose production and subsequent hyperglycemia. Additionally, corticosteroids reduce insulin sensitivity by impairing the action of insulin in peripheral tissues, particularly in muscle and adipose tissue. This impairment results in decreased glucose uptake and utilization, further contributing to elevated blood glucose levels [56,57,58].

The review of current literature shows a clear clinical connection between intra-articular corticosteroid injections and glucose levels, suggesting a likely impact on blood glucose levels following the administration of corticosteroids in any patient [59,60,61,62]. Studies indicate that this effect is likely more impactful in patients with poorly controlled diabetes or existing reduced insulin sensitivity. There appears to be conflicting evidence about the duration of impact the steroids have on glucose levels. Some studies show a return to baseline glucose levels two days following the administration of steroids, whereas others show a greater-than-expected increase in patients’ hemoglobin A1C months after injection. It was consistently seen throughout the various studies reviewed here that a return to baseline blood glucose was observed after a few months’ duration maximum.

The hyperglycemic effects of corticosteroids have profound clinical implications, particularly for patients with diabetes and less so for those patients at risk of developing the condition. For diabetic patients, the use of corticosteroids can exacerbate existing hyperglycemia, making blood glucose management more challenging. This transient period of uncontrolled blood sugar levels has a small chance of increasing the risk of diabetes-related complications such as neuropathy, nephropathy, and retinopathy, but may be less likely given the shorter duration of glucose fluctuations. The clinician should be aware, though, that frequent and recurrent corticosteroid injections to the peripheral joints may culminate in a lasting rise in glucose and increase the risk for end-organ damage related to diabetes. For patients at risk of developing diabetes, prolonged or repeated exposure to corticosteroids can also increase the likelihood of developing diabetes.

The clinical significance of these findings lies in the need for careful monitoring and management of blood glucose levels in patients receiving corticosteroid therapy. For diabetic patients, this may involve more frequent blood glucose monitoring and adjustments to their diabetes management plan to account for the hyperglycemic effects of corticosteroids. For patients at risk of developing diabetes, healthcare providers should consider the potential for steroid-induced diabetes and monitor blood glucose levels accordingly. Additionally, the findings underscore the importance of patient education, ensuring that patients are aware of the potential side effects of corticosteroids and the need for regular home blood glucose monitoring. In clinical practice, these insights can guide the development of comprehensive pain management plans that balance the benefits of corticosteroid therapy with the need to maintain glycemic control. Given the enlightening results of non-inferiority studies showing similar outcomes with lower injected steroid doses, the clinician should consider decreasing the amount of steroid used for injections while still achieving satisfactory patient outcomes. In addition, the effects on cortisol are poorly understood, though prevalent. Patients with poorly controlled diabetes that are at a higher risk of A1C derangement following injection should be considered for alternative therapies such as hyaluronic acid, peripheral nerve stimulation, extended-release steroid formulations, nerve blocks/ablations, bracing, and other management options.

A review of the existing literature provides many questions about the currently accepted approach to corticosteroid use in musculoskeletal and pain medicine. The tremendous heterogeneity in the patient population, agents used, their formulation, the routes of administration, and the various interventional targets preclude any definitive statements at this time. The evaluation of true risk for blood glucose elevation would require a large RTC controlling for specific patient populations, diagnoses, and with consistent agent and dosing protocols, with longer-term follow-up. This type of large study could potentially lead to better risk stratification or even a standardized scoring system to predict patient “fitness” for corticosteroid injection while avoiding complications and unnecessary burden and cost for patients and the hospital system. Furthermore, there are many unanswered questions about which factors might be predictive of said interventions causing corticosteroid-induced diabetes mellitus.

Futuristically, extended-release formulations of conventional steroids are currently approved for specific indications and, when studied, have been suggested not to cause significant alterations in the HPA or blood glucose levels [63]. One such example is Zilretta (triamcinolone acetonide extended-release injectable suspension), which is approved only for intra-articular injection in the knee [64]. It is hypothesized that any similar formulation could deliver the desired corticosteroid over a longer period at such a rate that it can alter local pathology and provide relief without the systemic burden discussed at length above. Further study is needed to evaluate these formulations’ safety and efficacy across a broader swath of interventional options, but this approach might open an avenue to more mindful and less deleterious interventional options, especially in diabetic patients.

It is also important to consider an approach for managing hyperglycemic episodes that may occur secondary to corticosteroid injection. While the individual burden of diabetes and subsequent response to corticosteroids varies, a clearly outlined approach to these complications can be modified as needed. The American Academy of Orthopedic Surgeons, American College of Sports Medicine, American Medical Society for Sports Medicine, and the American Diabetes Association make no recommendations for managing hyperglycemia in patients receiving steroid injections for musculoskeletal conditions [58]. Patients should be encouraged to consistently check their finger stick blood glucose at home. Any persistent hyperglycemia (blood glucose above 180 mg/dL) should prompt medical evaluation [65]. Close coordination with primary care or endocrinology may also be helpful in preparing patients to better manage any transient increases in blood glucose as well.

The clinical implications of these findings are profound. Transient hyperglycemia can pose challenges in managing diabetes and may elevate the risk of complications such as neuropathy, nephropathy, and retinopathy if glycemic control is not swiftly restored. Furthermore, the potential development of diabetic ketoacidosis can be a life-threatening complication. For non-diabetic patients, prolonged corticosteroid use may increase the risk of developing steroid-induced diabetes, necessitating careful patient selection and monitoring. Moreover, the systemic absorption of corticosteroids from intra-articular injections, though limited compared to oral or intravenous routes, still warrants caution, particularly in high-risk populations.

## 5. Conclusions

Corticosteroids remain a cornerstone in the management of inflammatory and musculoskeletal pain; however, their clinical utility is tempered by potential systemic side effects, particularly the impact on glucose metabolism [47,58,66]. The evidence reviewed highlights that corticosteroids can significantly elevate blood glucose levels through mechanisms such as enhanced gluconeogenesis, insulin resistance, and impaired glucose uptake. These effects are particularly pronounced in diabetic patients, with poorly controlled diabetes serving as a major risk factor for substantial glucose derangements [4,67,68]. To optimize patient outcomes, clinicians should adopt a tailored approach, emphasizing risk stratification, judicious corticosteroid use, and close monitoring of glucose levels pre- and post-injection [69]. The evaluation of diabetic patients in particular, prior to the clinical application of glucocorticoids, should include a baseline hemoglobin, preferably from within the preceding one to three months. The literature reviewed in this work suggests that an HbA1C above 7–8% would suggest a higher likelihood of inducing post-injection hyperglycemia, and such findings might warrant delaying intervention until the patient’s blood glucose levels are better controlled [43,48]. Alternative therapies, as mentioned above, should be considered for patients at high risk of glycemic complications. Primary care and endocrinology should be involved to help manage at-risk patients. Additionally, emerging evidence supporting the non-inferiority of lower corticosteroid doses for equivalent pain relief underscores the potential adoption of corticosteroid-sparing strategies.

In conclusion, while corticosteroids are invaluable in pain management, their metabolic effects necessitate a balanced approach that minimizes risks while maximizing therapeutic benefits. Educating patients and fostering collaborative management plans are pivotal steps in achieving this balance and ensuring safe, effective care.

## Data Availability

No new data were created or analyzed in this study. Data sharing is not applicable to this article.

## Abbreviations

The following abbreviations are used in this manuscript:

ADL	Activities of daily living
OA	Osteoarthritis
RA	Rheumatoid arthritis
NSAIDs	Nonsteroidal anti-inflammatory drugs
HbA1C	Hemoglobin A1c
COX-2	Cyclooxygenase-2
ESI	Epidural steroid injection
HPA	Hypothalamic–pituitary–adrenal

## References

[1] Hsu J.R., Mir H., Wally M.K., Seymour R.B., the Orthopaedic Trauma Association Musculoskeletal Pain Task Force (2019). Clinical Practice Guidelines for Pain Management in Acute Musculoskeletal Injury. J. Orthop. Trauma.

[2] Clauw D.J., Essex M.N., Pitman V., Jones K.D. (2019). Reframing chronic pain as a disease, not a symptom: Rationale and implications for pain management. Postgrad. Med..

[3] Derwich M., Mitus-Kenig M., Pawlowska E. (2021). Mechanisms of Action and Efficacy of Hyaluronic Acid, Corticosteroids and Platelet-Rich Plasma in the Treatment of Temporomandibular Joint Osteoarthritis—A Systematic Review. Int. J. Mol. Sci..

[4] Moon H.J., Choi K.H., Lee S.I., Lee O.J., Shin J.W., Kim T.W. (2014). Changes in Blood Glucose and Cortisol Levels After Epidural or Shoulder Intra-articular Glucocorticoid Injections in Diabetic or Nondiabetic Patients. Am. J. Phys. Med. Rehabil..

[5] Kapugi M., Cunningham K. (2019). Corticosteroids. Orthop. Nurs..

[6] MacMahon P.J., Eustace S.J., Kavanagh E.C. (2009). Injectable Corticosteroid and Local Anesthetic Preparations: A Review for Radiologists. Radiology.

[7] Yaftali N.A., Weber K. (2019). Corticosteroids and Hyaluronic Acid Injections. Clin. Sports Med..

[8] Freire V., Bureau N.J. (2016). Injectable Corticosteroids: Take Precautions and Use Caution. Semin. Musculoskelet. Radiol..

[9] Testa G., Giardina S.M.C., Culmone A., Vescio A., Turchetta M., Cannavò S., Pavone V. (2021). Intra-Articular Injections in Knee Osteoarthritis: A Review of Literature. J. Funct. Morphol. Kinesiol..

[10] Arroll B., Goodyear-Smith F. (2004). Corticosteroid injections for osteoarthritis of the knee: Meta-analysis. BMJ.

[11] DeFronzo R.A. (2004). Pathogenesis of type 2 diabetes mellitus. Med. Clin. N. Am..

[12] (2018). Essentials of Pain Medicine.

[13] Shah P., Kalra S., Yadav Y., Deka N., Lathia T., Jacob J.J., Kota S.K., Bhattacharya S., Gadve S.S., Subramanium K. (2022). Management of Glucocorticoid-Induced Hyperglycemia. Diabetes Metab. Syndr. Obes. Targets Ther..

[14] Nehme A., Zouein F.A., Zayeri Z.D., Zibara K. (2019). An Update on the Tissue Renin Angiotensin System and Its Role in Physiology and Pathology. J. Cardiovasc. Dev. Dis..

[15] Hall J.E., do Carmo J.M., da Silva A.A., Wang Z., Hall M.E. (2019). Obesity, kidney dysfunction and hypertension: Mechanistic links. Nat. Rev. Nephrol..

[16] Kufe D.W., Holland J.F., Frei E., American Cancer Society (2003). Cancer Medicine 6.

[17] Liu D., Ahmet A., Ward L., Krishnamoorthy P., Mandelcorn E.D., Leigh R., Brown J.P., Cohen A., Kim H. (2013). A practical guide to the monitoring and management of the complications of systemic corticosteroid therapy. Allergy Asthma Clin. Immunol..

[18] Brinks J., van Dijk E.H.C., Habeeb M., Nikolaou A., Tsonaka R., Peters H.A.B., Sips H.C.M., van de Merbel A.F., de Jong E.K., Notenboom R.G.E. (2018). The Effect of Corticosteroids on Human Choroidal Endothelial Cells: A Model to Study Central Serous Chorioretinopathy. Investig. Ophthalmol. Vis. Sci..

[19] Farman N., Rafestin-Oblin M.E. (2001). Multiple aspects of mineralocorticoid selectivity. Am. J. Physiol-Ren. Physiol..

[20] Ferrari P. (2010). The role of 11β-hydroxysteroid dehydrogenase type 2 in human hypertension. Biochim. Biophys. Acta BBA Mol. Basis Dis..

[21] Buyuk A.F., Kilinc E., Camurcu I.Y., Camur S., Ucpunar H., Kara A. (2017). Compared efficacy of intra-articular injection of methylprednisolone and triamcinolone. Acta Ortop. Bras..

[22] Centeno L.M., Moore M.E. (1994). Preferred intraarticular corticosteroids and associated practice: A survey of members of the American College of Rheumatology. Arthritis Rheum..

[23] Cushman D.M., Teramoto M., Asay A., Clements N.D., McCormick Z.L. (2021). Corticosteroid and Local Anesthetic Use Trends for Large Joint and Bursa Injections: Results of a Survey of Sports Medicine Physicians. PM&R.

[24] Valtonen E.J. (1981). Clinical comparison of triamcinolonehexacetonide and betamethasone in the treatment of osteoarthrosis of the knee-joint. Scand. J. Rheumatol. Suppl..

[25] Pyne D., Ioannou Y., Mootoo R., Bhanji A. (2004). Intra-articular steroids in knee osteoarthritis: A comparative study of triamcinolone hexacetonide and methylprednisolone acetate. Clin. Rheumatol..

[26] Stone S., Malanga G.A., Capella T. (2021). Corticosteroids: Review of the History, the Effectiveness, and Adverse Effects in the Treatment of Joint Pain. Pain Physician..

[27] Park S.K., Choi Y.S., Kim H.J. (2013). Hypopigmentation and subcutaneous fat, muscle atrophy after local corticosteroid injection. Korean J. Anesthesiol..

[28] Shah A., Mak D., Davies A.M., James S.L., Botchu R. (2019). Musculoskeletal Corticosteroid Administration: Current Concepts. Can. Assoc. Radiol. J..

[29] Gray R.G., Tenenbaum J., Gottlieb N.L. (1981). Local corticosteroid injection treatment in rheumatic disorders. Semin. Arthritis Rheum..

[30] Conn D.L. (2001). Resolved: Low-dose prednisone is indicated as a standard treatment in patients with rheumatoid arthritis. Arthritis Rheum..

[31] Ramamoorthy S., Cidlowski J.A. (2016). Corticosteroids. Rheum. Dis. Clin. N. Am..

[32] Yu S., Meng S., Xiang M., Ma H. (2021). Phosphoenolpyruvate carboxykinase in cell metabolism: Roles and mechanisms beyond gluconeogenesis. Mol. Metab..

[33] Beaupere C., Liboz A., Fève B., Blondeau B., Guillemain G. (2021). Molecular Mechanisms of Glucocorticoid-Induced Insulin Resistance. Int. J. Mol. Sci..

[34] Fujimoto W.Y. (2000). The importance of insulin resistance in the pathogenesis of type 2 diabetes mellitus. Am. J. Med..

[35] Schäcke H., Döcke W.D., Asadullah K. (2002). Mechanisms involved in the side effects of glucocorticoids. Pharmacol. Ther..

[36] Williams D.M. (2018). Clinical Pharmacology of Corticosteroids. Respir. Care.

[37] Lambillotte C., Gilon P., Henquin J.C. (1997). Direct glucocorticoid inhibition of insulin secretion. An in vitro study of dexamethasone effects in mouse islets. J. Clin. Investig..

[38] Campos C. (2012). Chronic Hyperglycemia and Glucose Toxicity: Pathology and Clinical Sequelae. Postgrad. Med..

[39] Stout A., Friedly J., Standaert C.J. (2019). Systemic Absorption and Side Effects of Locally Injected Glucocorticoids. PM&R.

[40] Kamel S.I., Rosas H.G., Gorbachova T. (2024). Local and Systemic Side Effects of Corticosteroid Injections for Musculoskeletal Indications. AJR Am. J. Roentgenol..

[41] Liu X.-X., Zhu X.-M., Miao Q., Ye H.-Y., Zhang Z.-Y., Li Y.-M. (2014). Hyperglycemia Induced by Glucocorticoids in Nondiabetic Patients: A Meta-Analysis. Ann. Nutr. Metab..

[42] Abdul A.J., Ghai B., Bansal D., Sachdeva N., Bhansali A., Dhatt S.S. (2017). Hypothalamic Pituitary Adrenocortical Axis Suppression following a Single Epidural Injection of Methylprednisolone Acetate. Pain. Physician.

[43] Hao C., Yin J., Liu H., Cheng Z., Zeng Y., Jin Y. (2024). Pain Reduction and Changes in Serum Cortisol, Adrenocorticotropic Hormone, and Glucose Levels after Epidural Injections with Different Doses of Corticosteroid. Pain. Physician.

[44] Mader R., Lavi I., Luboshitzky R. (2005). Evaluation of the pituitary–adrenal axis function following single intraarticular injection of methylprednisolone. Arthritis Rheum..

[45] Lamer T.J., Dickson R.R., Gazelka H.M., Nicholson W.T., Reid J.M., Moeschler S.M., Hooten W.M. (2018). Serum Triamcinolone Levels following Cervical Interlaminar Epidural Injection. Pain Res. Manag..

[46] Hooten W.M., Nicholson W.T., Gazelka H.M., Reid J.M., Moeschler S.M., Lamer T.J. (2016). Serum Triamcinolone Levels Following Interlaminar Epidural Injection. Reg. Anesth. Pain Med..

[47] Choudhry M.N., Malik R.A., Charalambous C.P. (2016). Blood Glucose Levels Following Intra-Articular Steroid Injections in Patients with Diabetes: A Systematic Review. JBJS Rev..

[48] Sytsma T.T., Greenlund L.S., Fischer K.M., McCoy R.G. (2024). Impact of Intra-Articular Corticosteroid Injection on Glycemic Control: A Population-Based Cohort Study. Clin. Diabetes.

[49] Park J., Kwak J., Chung S., Hong H.J., Chon J.Y., Moon H.S. (2020). Incidence of Adrenal Insufficiency and Cushing’s Syndrome After Long-Term Epidural Steroid Injections Over Six Months or Longer: A Preliminary Study. J. Pain Res..

[50] Friedly J.L., Comstock B.A., Heagerty P.J., Bauer Z., Rothman M.S., Suri P., Hansen R., Avins A.L., Nedeljkovic S.S., Nerenz D.R. (2018). Systemic effects of epidural steroid injections for spinal stenosis. Pain.

[51] Utamawatin K., Phruetthiphat O.A., Apinyankul R., Chaiamnuay S. (2023). The efficacy of intra-articular triamcinolone acetonide 10 mg vs. 40 mg in patients with knee osteoarthritis: A non-inferiority, randomized, controlled, double-blind, multicenter study. BMC Musculoskelet. Disord..

[52] Onks C., Weaver L., Latorre J., Silvis M., Berg A., Phillips S., Loeffert J., French C., Armstrong A. (2024). The most effective corticosteroid dose in the treatment of glenohumeral osteoarthritis: Feasibility pilot and protocol for double blinded randomized controlled trial. Osteoarthr. Cartil. Open.

[53] Chow R., Ng J., Wood M., Yanez D., He Z., Rajput K. (2025). A comparison of steroid dose with or without local anesthetic in lumbar interlaminar epidural steroid injections. Pain. Pract..

[54] Quatrini L., Ugolini S. (2021). New insights into the cell- and tissue-specificity of glucocorticoid actions. Cell. Mol. Immunol..

[55] Ingawale D.K., Mandlik S.K. (2020). New insights into the novel anti-inflammatory mode of action of glucocorticoids. Immunopharmacol. Immunotoxicol..

[56] Perez A., Jansen-Chaparro S., Saigi I., Bernal-Lopez M.R., Miñambres I., Gomez-Huelgas R. (2014). Glucocorticoid-induced hyperglycemia. J. Diabetes.

[57] Hwang J.L., Weiss R.E. (2014). Steroid-induced diabetes: A clinical and molecular approach to understanding and treatment. Diabetes Metab. Res. Rev..

[58] Waterbrook A.L., Balcik B.J., Goshinska A.J. (2017). Blood Glucose Levels After Local Musculoskeletal Steroid Injections in Patients With Diabetes Mellitus: A Clinical Review. Sports Health Multidiscip. Approach.

[59] Stepan J.G., London D.A., Boyer M.I., Calfee R.P. (2014). Blood glucose levels in diabetic patients following corticosteroid injections into the hand and wrist. J. Hand Surg..

[60] Mathieu S., Couderc M., Malochet-Guinamand S., Dubost J.J., Tournadre A., Soubrier M. (2019). Effect of Corticosteroid Injections on Blood Glucose Levels in Diabetic Patients. JCR J. Clin. Rheumatol..

[61] Hoshino R., Wakaizumi K., Otsuka Y., Morisaki H., Kosugi S. (2023). Effect of repeated transforaminal epidural low-dose dexamethasone injections on glucose profiles of diabetic and non-diabetic patients with low back pain. J. Anesth..

[62] Aleem A.W., Syed U.A.M., Nicholson T., Getz C.L., Namdari S., Beredjiklian P.K., A Abboud J. (2017). Blood Glucose Levels in Diabetic Patients Following Corticosteroid Injections into the Subacromial Space of the Shoulder. Arch. Bone Jt. Surg..

[63] Russell S.J., Sala R., Conaghan P.G., Habib G., Vo Q., Manning R., Kivitz A., Davis Y., Lufkin J., Johnson J.R. (2018). Triamcinolone acetonide extended-release in patients with osteoarthritis and type 2 diabetes: A randomized, phase 2 study. Rheumatology.

[64] Kraus V., Conaghan P., Aazami H., Mehra P., Kivitz A., Lufkin J., Hauben J., Johnson J., Bodick N. (2018). Synovial and systemic pharmacokinetics (PK) of triamcinolone acetonide (TA) following intra-articular (IA) injection of an extended-release microsphere-based formulation (FX006) or standard crystalline suspension in patients with knee osteoarthritis (OA). Osteoarthr. Cartil..

[65] Moghissi E.S., Korytkowski M.T., DiNardo M., Einhorn D., Hellman R., Hirsch I.B., Inzucchi S.E., Ismail-Beigi F., Kirkman M.S., Umpierrez G.E. (2009). American Association of Clinical Endocrinologists and American Diabetes Association consensus statement on inpatient glycemic control. Diabetes Care.

[66] Habib G.S., Bashir M., Jabbour A. (2008). Increased blood glucose levels following intra-articular injection of methylprednisolone acetate in patients with controlled diabetes and symptomatic osteoarthritis of the knee. Ann. Rheum. Dis..

[67] Younes M., Neffati F., Touzi M., Hassen-Zrour S., Fendri Y., Béjia I., Ben Amor A., Bergaoui N., Najjar M.F. (2007). Systemic effects of epidural and intra-articular glucocorticoid injections in diabetic and non-diabetic patients. Jt. Bone Spine.

[68] Kallock E., Neher J.O., Safranek S. (2010). Clinical inquiries. Do intra-articular steroid injections affect glycemic control in patients with diabetes?. J. Fam. Pract..

[69] Nguyen V.H., Goel A.P., Yerra S., Hamill-Ruth R. (2018). Use of a Screening Questionnaire to Identify Patients at Risk of Hyperglycemia Prior to Steroid Injection Therapy. Pain Med..

